# *Orientia tsutsugamushi* in Eschars from Scrub Typhus Patients

**DOI:** 10.3201/eid1207.050827

**Published:** 2006-07

**Authors:** Yun-Xi Liu, Wu-Chun Cao, Yuan Gao, Jing-Lan Zhang, Zhan-Qing Yang, Zhong-Tang Zhao, Janet E. Foley

**Affiliations:** * Beijing Institute of Microbiology and Epidemiology, Beijing, People's Republic of China;; † Center for Disease Control and Prevention of Jinan, Jinan, People's Republic of China;; ‡ Shandong University, Jinan, People's Republic of China;; § University of California, Davis, Davis, California, USA

**Keywords:** Scrub typhus, eschar, Orientia tsutsugamushi

## Abstract

To verify the value of eschars for the diagnosis of scrub typhus and to characterize genotypes of *Orientia tsutsugamushi* in patients, we examined eschars and blood specimens of 7 patients from Shandong Province, People's Republic of China, for *O. tsutsugamushi* by polymerase chain reaction targeting the Sta56 gene. All 7 eschars and acute-phase blood samples were positive, while no specific DNA amplicons were obtained from the 7 convalescent-phase blood samples collected after antimicrobial drug therapy. The findings indicate that patients' eschars can be used for detection and genetic characterization of *O. tsutsugamushi* during the convalescent phase.

## Abstract 2

Eschars can be used for genetic characterization of *O. tsutsugamushi* during the convalescent phase.

Scrub typhus, a widely endemic disease in Asian Pacific regions, is caused by *Orientia tsutsugamushi*, a gram-negative obligate intracellular bacterium in the family *Rickettsiaceae*. When the rickettsia is transmitted to a human through the bite of an infected mite, it begins to multiply at the bite site, and a characteristic skin lesion known as an eschar is formed. The pathogen then spreads systemically by the hematogenous and lymphogenous routes. Various clinical manifestations develop, including fever, rash, and lymphadenopathy (*1*).

Before 1986, scrub typhus was only found in southern China (south of the Yangtze River) primarily in the summer. *O*. *tsutsugamushi*, which causes "summer-type scrub typhus," is highly virulent and usually transmitted by the *Leptotrombidium deliense* mite. In 1986, scrub typhus was first reported in Mengyin County, Shandong Province, north of the Yangtze River. This newly recognized "autumn-winter type scrub typhus" is caused by a less virulent strain of *O*. *tsutsugamushi* and transmitted by the *L*. *scutellare* mite (*1**,**2*). Since then, cases of autumn-winter scrub typhus have been increasingly reported in many northern areas of China; eschars developed in 82%–91% of those infected (*1**,**2*).

Traditionally, the diagnosis of scrub typhus mainly relied on serologic tests. The disease could be retrospectively diagnosed in cases of seroconversion or a >4-fold rise in antibody titers between acute-phase and convalescent-phase serum specimens. The requirement of double serum specimens has limited its usage for diagnosis. Recently a polymerase chain reaction (PCR) assay was developed for detecting *O*. *tsutsugamushi* Sta56 gene in blood samples or isolates from patients (*3**–**8*). However, the test often gave a false-negative result because hemoglobin and other components in blood may inhibit PCR amplification (*3**,**4**,**9*). The commonly seen eschars in scrub typhus patients were suggested as alternative specimens for diagnosis (*9*). The objectives of this study were to verify the value of eschars for the diagnosis of scrub typhus by PCR assay and to characterize the genotype of *O*. *tsutsugamushi* during the convalescent phase.

## Materials and Methods

### Sample Collection

Seven scrub typhus patients reported at Feixian County (116°11´–118°18´E, 35°01´–35°33´N), Shandong Province, China, in September, October, and November of 2003–2004 were included in the study. The identification (ID) codes, age, sex, the locations of eschars, and other clinical characteristics on admission were documented (Table). The typical eschars of 2 patients (03PE1 and 04PE5) are shown in the Figure. After informed consent was obtained, 5 mL acute-phase blood was collected from each patient before treatment. Chloramphenicol was then administered orally at a dosage of 1.5–2.5 g 4×/day for 4–5 days. Fever resolved for all 7 patients within 2 days of treatment. Eschar specimens and 5-mL convalescent-phase blood sample from each patient were collected at the time that the eschar spontaneously desquamated (6–15 days after treatment). Serum specimens were separated by centrifugation at 2,500× *g* for 10 min. All specimens from eschars, serum, and residual blood clots were kept at -70°C until use.

**Table:** Clinical characteristics on admission and serologic results of 7 patients with scrub typhus*

Patient ID	Age, y (sex)	Fever, °C	Rash	Lymphadenitis	Location of eschars	Acute phase		Convalescent phase	
						Blood samples collected, d after onset	IgG titers of sera	Eschars and blood samples collected,† d after onset	IgG titers of sera
03PE1	35 (F)	38	+	+	Neck	3	N	9	320
03PE2	42 (M)	40	+	+	Umbilicus	7	80	8	320
03PE3	48 (M)	40	+	+	Right groin	5	N	10	320
03PE4	28 (F)	38	+	+	Left papilla	8	160	11	640
04PE5	53 (M)	38	+	+	Waist	2	N	6	320
04PE6	36 (M)	40	+	+	Behind right ear	10	80	15	1,280
04PE7	31 (F)	39	+	+	Left axilla	7	N	9	640

*Ig, immunoglobulin; N, negative. †Chloramphenicol was given to a patient immediately after the acute-phase blood was collected.

**Figure:**
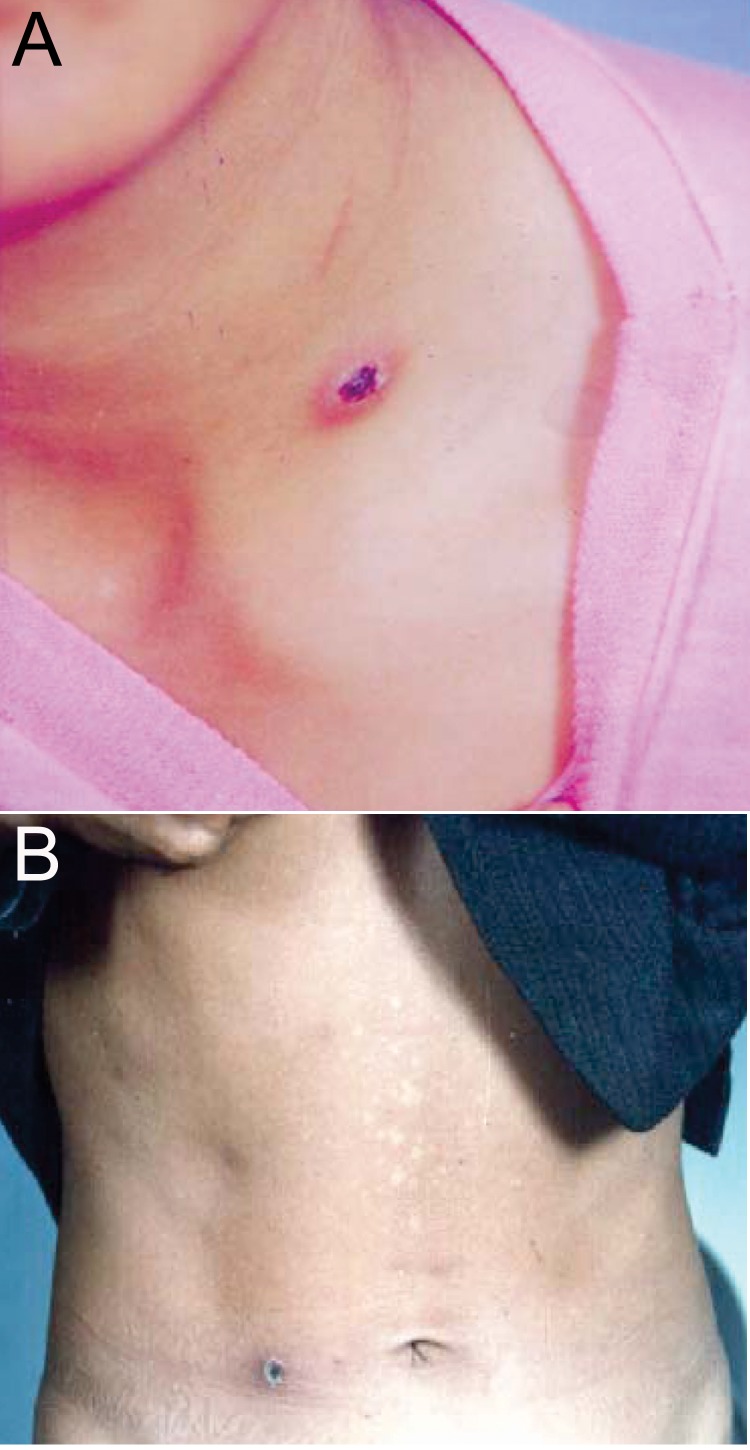
The locations of typical eschars in 2 representative patients with scrub typhus. A) An eschar on the neck of a patient (03PE1). B) An eschar on the waist of a patient (04PE5).

### Detection of IgG Antibodies against *O. tsutsugamushi*

An indirect immunofluorescent antibody assay (IFA) was performed as described previously (*10*), by using mixed Gilliam, Karp, and Kato strains of *O*. *tsutsugamushi* as diagnostic antigen. Scrub typhus was diagnosed in the case of seroconversion or a >4-fold rise in IgG antibody titers between acute-phase and convalescent-phase sera.

### DNA Extraction

Complete eschars (30–60 mg in weight) or 0.3 mL blood clot was homogenized with TE (10 mmol/L Tris Cl [pH 8.0] and 1 mmol/L EDTA) buffer and centrifuged at 3,000× *g* for 5 min; the supernatant was discarded. For the blood clot, the precipitate was resuspended and washed with TE buffer 3 times to eliminate the residual inhibitors in blood. Then 400 μL lysis buffer (10 mmol/L Tris [pH 8.0], 0.1 mol/L EDTA, 0.5% sodium dodecyl sulfate), 10 μL proteinase K (20 mg/mL; Promega Corp., Madison, WI, USA), and 2 μL lysozyme (4 mg/mL; DingGuo Biotech. Co. Ltd, Beijing, People's Republic of China) were added, and incubated at 50°C for 6 h. DNA was extracted with phenol/chloroform/isoamylalcohol (25:24:1) and precipitated in ethanol. Finally, the DNA was washed with 75% ethanol and dissolved in 20 μL distilled water.

### PCR Amplification

PCR amplification of the Sta56 gene was performed by using species-specific primers, Pr1 (5´-tac att agc tgc agg tat gac-3´) and Pr2 (5´-AAT TCT TCA ACC AAG CGA TCC-3´) (*3**,**4**,**10*). The amplifications were performed in a volume of 50 μL with a Perkin-Elmer model 2400 thermal cycler (Perkin-Elmer, Norwalk, CT, USA). The amplification program consisted of 1 cycle for 5 min at 94°C, 35 cycles of denaturation at 94°C for 30 s, annealing at 56°C for 1 min, and extension at 72°C for 1 min. This process was followed by a final extension at 72°C for 5 min. The amplification products then underwent electrophoresis in a 2% agarose gel containing ethidium bromide and visualized under UV light.

The acute- and convalescent-phase blood samples were processed and run through the PCR instrument at the same time as the eschar specimens. DNA from reference strains of Gilliam, Karp, and Kato was used as positive controls, and distilled water was used as a negative control in each amplification. To avoid contamination, DNA extraction, reagent setup, PCR, and electrophoresis were performed in separate rooms.

### Sequence Analysis

The purified PCR amplicons of all the positive samples were sequenced by Shanghai Invitrogen Biotechnology Co. Ltd (Shanghai, People's Republic of China). The sequences were compared with all available reported *O. tsutsugamushi* Sta56 gene sequences in GenBank by using BLAST (Basic Local Alignment Search Tool) program (available from http://www.ncbi.nlm.nih.gov/BLAST). The GenBank accession numbers of sequences obtained from the 7 patients in this study are DQ188085, DQ188086, DQ188087, DQ188088, DQ188089, DQ188090, and DQ188091, respectively.

## Results

Seroconversion or a >4-fold rise in titers of IgG antibody to *O. tsutsugamushi* was observed in all 7 patients (Table), thus confirming the diagnosis of scrub typhus. Seven eschars and 7 acute-phase blood samples from the patients were positive by PCR targeting the Sta56 gene, while 7 convalescent-phase blood samples collected after antimicrobial drug treatment were all PCR-negative.

The 317-bp partial sequence of the *O. tsutsugamushi* Sta56 gene amplified from each eschar was identical to that of its corresponding acute-phase blood sample. The sequences from the 7 patients differed from each other by 1 or 2 bp; two sequences were identical. The nucleotide sequences were 95.6%–97.8% homologous with the corresponding parts of Kawasaki strain Sta56 gene deposited in GenBank (accession no. M63383), while the sequence homologies with other strains such as Karp, Kato, Kuroki, Shimokoshi, and Je-cheon were all <75.87%.

## Discussion

Previous studies used spleen tissues of infected mammals or acute-phase blood from patients to detect *O. tsutsugamushi* by PCR (*3**,**4*). However, PCR amplification of *O. tsutsugamushi* DNA from blood often lacks sensitivity because some hemoglobin, iron porphyrin, and other factors may inhibit the PCR, although obtaining and processing the blood that avoids the inhibitors is possible (*3**,**4**,**9*). Ono et al. previously found *O. tsutsugamushi* DNA (identified as Kawasaki type) in only 1 patient's eschar before antimicrobial drug treatment but not in the acute-phase blood sample (*9*). In the present study, 7 scrub typhus patients were examined, and *O. tsutsugamushi* DNA was successfully detected in their spontaneously desquamated eschars and acute-phase blood samples. These findings further proved that eschars could be used as an alternative, easily acquired, and sensitive sample for the diagnosis of *O. tsutsugamushi* infection, particularly when persons are reluctant to provide a blood sample because of cultural or other reasons.

We described a new simple confirmatory diagnostic assay in which eschars are used as an alternative to serologic tests such as IFA, which usually requires double blood samples from acute and convalescent phases. In addition, from the successful and efficient detection of the *O. tsutsugamushi* DNA in naturally desquamated eschars, we can infer the presence of the agent in eschars before beginning antimicrobial drug therapy. If eschars had been sampled during the acute phase by punch biopsy, this method could be used for the early diagnosis of scrub typhus.

A previous study carried out in Thailand detected *O. tsutsugamushi* DNA in convalescent-phase blood of patients after a single dose of doxycycline (*10*). However, in the present study, *O. tsutsugamushi* DNA was not persistent in the convalescent-phase blood of patients after 4–5 days of chloramphenicol treatment. Whether a lack of PCR sensitivity or difference in the treatment regimens explains the apparent lack of *O. tsutsugamushi* DNA in the convalescent-phase blood samples is not known. A possible reason is that the patients in the present study were infected with the less virulent strain of *O. tsutsugamushi* (*11*), which may only persist in blood for a short period after antimicrobial drug treatment. Our previous study indicated that to isolate *O. tsutsugamushi* from patients with autumn-winter scrub typhus, cyclophosphamide (0.25 mg/g of body weight) had to be injected into the experimental mice after injection of patients' blood to suppress immunity (*11*).

Sequence analysis of partial Sta56 gene clarified that the genotypes of *O. tsutsugamushi* in the scrub typhus patients from Shandong Province, China, were more closely related to Kawasaki type, which is less virulent than other genotypes and only caused a mild syndrome (*1*). The finding has applications for physicians to treat patients and prescribe medicine.
